# A Novel Technique Using Polytetrafluoroethylene Tape to Solve Screw Loosening Complication in Implant-Supported Single Crowns

**DOI:** 10.3390/ijerph18010125

**Published:** 2020-12-27

**Authors:** Luis F. Félix, Michell Medina, Cristina Gómez-Polo, Rubén Agustín-Panadero, Rocío Ortega, Miguel Gómez-Polo

**Affiliations:** 1 Department of Prosthodontics and Restorative Dentistry, School of Dentistry, Complutense University of Madrid, 28040 Madrid, Spain; michellmd89@gmail.com; 2 Department of Surgery, Faculty of Medicine, University of Salamanca, 37007 Salamanca, Spain; crisgodent@hotmail.com; 3 Department of Stomatology, Faculty of Medicine and Dentistry, University of Valencia, 46010 Valencia, Spain; rubenagustinpanadero@gmail.com; 4 Department of Prosthetic Dentistry, School of Dentistry, European University of Madrid, 28045 Madrid, Spain; rocio.ortega@universidadeuropea.es; 5 Department of Prosthetic Dentistry, Faculty of Dentistry, Complutense University of Madrid, 28040 Madrid, Spain; miguelodont@hotmail.com

**Keywords:** screw loosening, implant single crowns, fixed dental prosthesis, polytetrafluorethylene, mechanical complications

## Abstract

This study aimed to analyze a novel technique to make screws with greater untightening resistance and to solve screw loosening in implant-supported single crowns. Thirty grade IV titanium straight abutments were screwed onto 30 external hex implants using grade IV titanium screws (30 Ncm). They were exposed to cyclic loading (300,000 cycles, 200 N). Samples were divided into 4 groups (15 samples per group): new screws (SCREW group) (control), reused screws (rSCREW group), new screws wrapped with polytetrafluoroethylene (PTFE) tape (PTFE group), and reused screws wrapped with PTFE tape (rPTFE group). Reverse torque values (RTVs) were recorded with a digitally calibrated implant motor. Mean RTVs observed were 14.46 N (±1.10 N) for the control group, 14.42 N (±1.22 N) for the rSCREW group, 19.97 N (±1.16 N) for the PTFE group, and 19.13 N (±2.38 N) for the rPTFE group. Statistically significant differences were found between RTVs of both groups employing screws without PTFE tape (SCREW and rSCREW groups) compared with those using screws wrapped with PTFE tape (PTFE and rPTFE) (*p* < 0.001). These results suggest that wrapping the implant–abutment screw with PTFE tape may effectively lower the risk of loosening and even constitute a solution when this complication occurs in implant single crowns.

## 1. Introduction

Implant-supported fixed dental prostheses (FDPs) are the alternative of choice to replace lost teeth, from a single crown to a complete arch [1,2,3,4,5]. 

Over time, dental implants have undergone considerable progress and change in design to reduce the number and severity of the associated biological and mechanical complications [6]. Nevertheless, screw loosening (SL) has been reported as one of the most common mechanical complications [7,8,9], with incidence ranging from 5% to 43% after the first year after placement [10,11,12,13]; these values denote wide variability in the clinical findings reported in articles on the subject. Such differences may be attributable to the broad spectrum of factors involved, which, among others, include the type of connection, type of prosthesis (single crown, partial or full-arch FDP), force magnitude and angle, and parafunctional habits. Complications with implant prostheses can cause physical, social, and psychological issues for the patient, leading to additional time and cost. Patients’ perception of the prosthetic outcome can affect their satisfaction with their dental treatment, their oral-health-related quality of life (OHRQoL), and ultimately, their general health [14]. 

SL has been described as a two-stage development [15], beginning with the onset of external loads that reduce screw preload, inducing vibration and micromovements. When preload subsequently drops below a critical value, the external forces and vibrations cause the implant–abutment screw to turn or recede, inducing SL. These micromovements may ultimately lead to biological complications due to the ingress of microorganisms that can destroy the bone surrounding the implant [16].

A number of studies have been conducted to determine the factors affecting SL, comparing implant connections, applying lateral loads, or varying the abutment angle or tightening conditions [17,18,19]. The conclusion drawn from the findings is that all may have an impact and that the etiology is therefore multifactorial. The approaches proposed to lower the frequency of SL may be divided into three groups. First, related to prosthetic design, includes centering occlusal contact, narrowing the buccolingual width of the crown, or shortening the cantilever. Second, involves tightening techniques, such as ensuring the right torque when tightening screws, the use of straight abutments, or screw retightening during placement. Third, screw design, which entails manufacturing screws from different materials or coating them with a material not used in their composition, such as steel or gold [20,21,22,23,24,25,26]. None of those methods seems to wholly prevent the appearance of this complication, however, because, given the multifactorial nature of the cause, a single solution for all circumstances would appear to be elusive [27].

In light of the foregoing and given the significance of SL in the long-term success of implant-supported FDP, more research is needed to find alternatives not only to reduce the incidence of screw loosening but also to correct it when it arises. One of the techniques addressed in earlier studies is to sheathe the screw in a material such as gold that would raise the reverse torque value. That approach has proven to be only partially effective, however, and very costly [18]. To date, no material has been described that would be applicable by both the manufacturer in new screw design and clinicians when patients present with loose prostheses. 

One of the materials that might be used to that end, given its biocompatibility, resistance to chemical agents, water repellence, ready adaptability, and durability, is polytetrafluorethylene (PTFE) tape, used in dentistry for other purposes [28,29,30]. Previous studies carried out have revealed that the use of lubricants like saliva could reduce friction and consequently increase the preload [31,32,33].

To the authors’ knowledge, no comparative data are available regarding the effect of wrapping abutment screws in PTFE tape to lower the risk of screw loosening in single-crown implants. This in vitro study explored wrapping screws in PTFE as a possible approach to manufacturing screws with greater untightening resistance and solving SL-associated clinical problems. The efficacy of the method was measured in terms of the removal torque value (RTV) in abutments screwed to external hexagon connection implants. External hexagon implants were intentionally chosen, as their design is where screws have greater influence on SL [34]. The null hypothesis was that differences would be observed between the RTVs for the control and experimental groups in the SL.

## 2. Materials and Methods 

**Figure 1:**
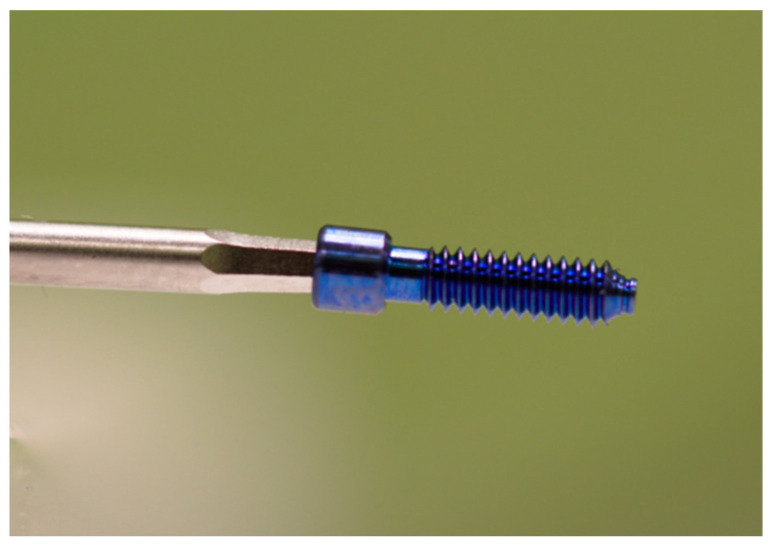
Original grade IV titanium prosthetic screw.

Thirty prefabricated milled titanium (grade IV) abutments (straight abutment Osseous STD; TiCare^®^, Valladolid, Spain) were fixed onto 30 implants (Osseous STD 3.75 × 11.5 mm; TiCare^®^, Valladolid, Spain) with a 4.1 mm platform diameter and external hexagonal connection (2.7 × 0.7 mm) implants. Thirty original screws (grade IV titanium) (prosthetic screws; TiCare^®^, Valladolid, Spain) were employed for all the implant–abutment specimens (Figure 1). The abutment screws had a hexagonal socket head. Samples were divided into 4 groups: SCREW group (control group; *n* = 15) (new screws), rSCREW group (reused screws without PTFE tape; *n* = 15), PTFE group (new screws wrapped with polytetrafluorethylene tape (MIARCO^®^, COD 269, 12 mm × 12 m × 0.1 mm, Valencia, Spain) (PTFE); *n* = 15), and rPTFE group (reused screws wrapped with PTFE tape; *n* = 15). The abutments were tightened using an implant motor (iChiroPro^®^; BienAir, Bienne, Switzerland) connected to a tablet computer (iPad; Apple Inc, California, CA, USA). This motor was managed with an application (iChiroPro APP v2.2; Bien Air, Bienne, Switzerland) where torque was previously set. The screws of all the groups were tightened clockwise to a torque of 30 Ncm pursuant to manufacturer’s instructions. This device was previously validated employing an Instron testing machine (Instron Corp, Norwood, Mass, model 4204, Barcelona, Spain) by a specialized engineer in order to ensure its reliability.

**Figure 2:**
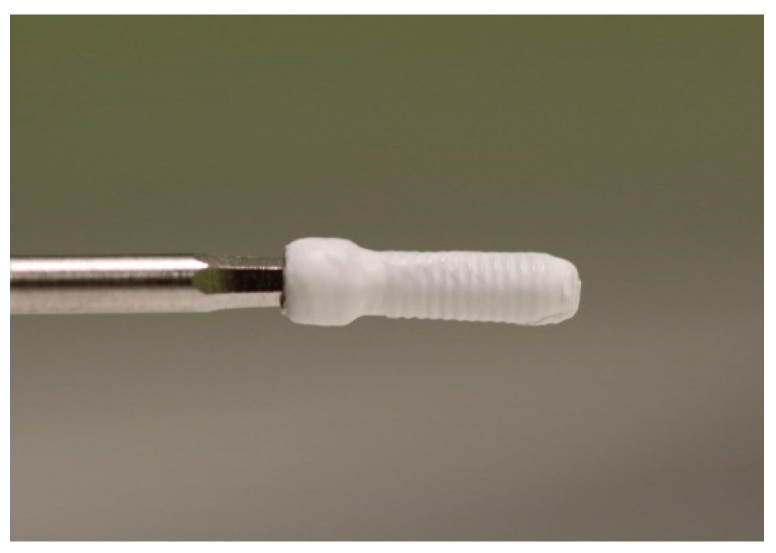
Prosthetic screw wrapped in polytetrafluorethylene (PTFE).

Four different techniques were employed for the screw tightening. For the control group (SCREW group), all the steps were carried out following the manufacturer’s instructions. New screws were used with no PTFE and in a dry environment. For the experimental PTFE group, PTFE tape (12 mm wide, 100 μm thick) was wound around the screws three times, and the PTFE tail at the bottom of the screw was subsequently cut away (Figure 2). The screw was tightened using a 1.25 hexagonal point connected to an implant motor (iChiroPro^®^; BienAir, Bienne, Switzerland) and was programmed at a low speed (12 revolutions per minute (rpm)). Then, the PTFE tape edge was put in contact with the screw and the implant motor was activated for 15 s so the screw was wound three times, therefore wrapping the screw in three layers of PTFE. 

**Figure 3:**
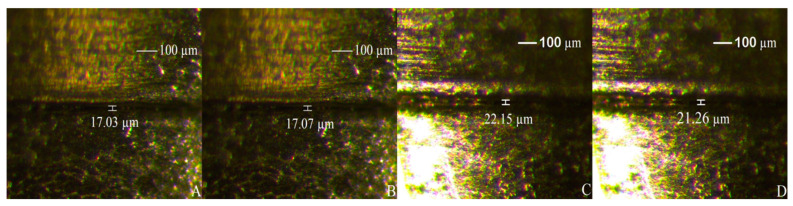
Implant–abutment misfit: SCREW (control (**A**)) specimen with original prosthetic screws and rSCREW group (**B**). PTFE (**C**) and rPTFE group (**D**) specimen with screws wrapped in PTFE tape.

The same methodology from the SCREW and PTFE groups was developed for the rPTFE and rSCREW groups. It is necessary to highlight that the screws employed in the SCREW and PTFE groups were respectively reused for the experimental rSCREW and rPTFE groups, so all the screws were previously loaded to 1 year of simulated mastication (300,000 cycles, 200 N) and intentionally unscrewed. It must be highlighted that the reused screws of rPTFE specimens were wrapped again with 3 layers of PTFE tape. A stereomicroscope (Leica M80, Wetzlar, Germany) at 60× magnification was used in order to visually determine if the use of PTFE tape created a gap between the implant and implant abutment. All the specimens were evaluated taking 4 measurements (1 per aspect) (Mesial, Distal, Vestibular and Lingual) by 1 blinded examiner. No changes were found (Figure 3).

**Figure 4:**
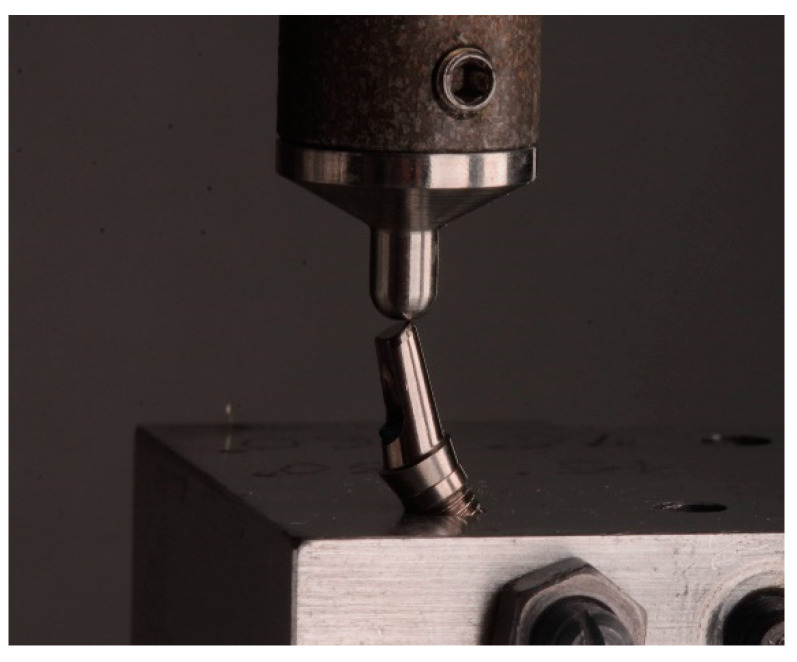
Cyclic loading following ISO 14801.

Each sample was positioned on a frame with the implant angulated 30° to load the abutment relative to the implant following the ISO 14801 normative. A force of 200 N was applied at a frequency of 2 Hz for 300,000 cycles, equivalent to 1 year of mastication [35] using a cyclic loading machine (Chewing Simulator CS-4.2 economy line^®^; SD Mechatronik GMBH, Feldkirchen-Westerham, Germany) (Figure 4).

**Figure 5:**
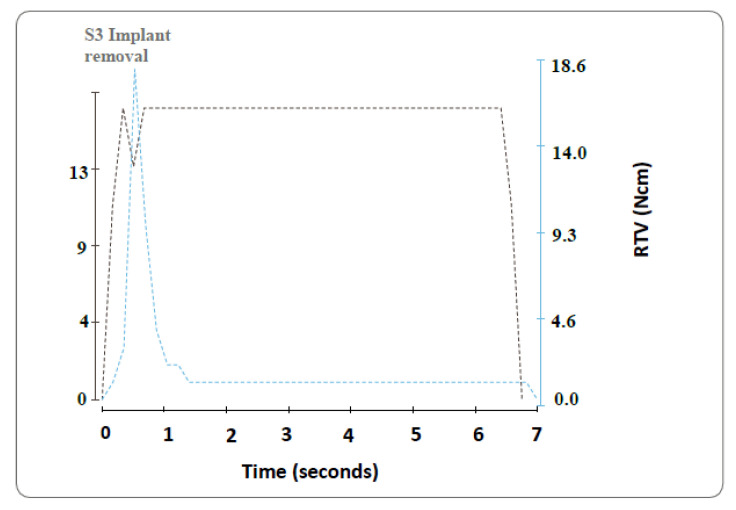
Example of reverse torque value (RTV) evolution during the untightening procedure.

Abutment tightening and recording of the RTVs for all the specimens were carried out with the same validated device used previously in their tightening (iChiroPro; Bien Air, California, CA, USA). The device managed by this software (iChiroPro APP v2.2; Bien Air, Bienne, Switzerland) can not only obtain the RTV but also create a graphic showing its evolution (Figure 5). For the analysis of power for 4 groups (*n* = 15), the Neyman–Pearson lemma was performed for a normalized size of the samples after assuming the risk of 0.2 and a power of 0.8, resulting in 11.44 samples per group. Finally, a greater sample size was included (*n* = 15). All data were statistically analyzed by Kolmogorov–Smirnov and Shapiro–Wilk tests. These were done to test normality and homogeneity variances of the data. Pairwise comparisons by Tukey’s test compared the RTVs between the different groups at a 95% significance level.

## 3. Results

The Shapiro–Wilk test for normal distribution was conducted on all four groups. All the groups showed values higher than the *p* = 0.05 Shapiro–Wilk criterion. The null hypothesis held and the samples studied could be deemed to be normally distributed (*p* = 0.448 SCREW group, *p* = 0.214 rSCREW group, *p* = 0.863 PTFE group, and *p* = 0.471 rPTFE group).

**Figure 6:**
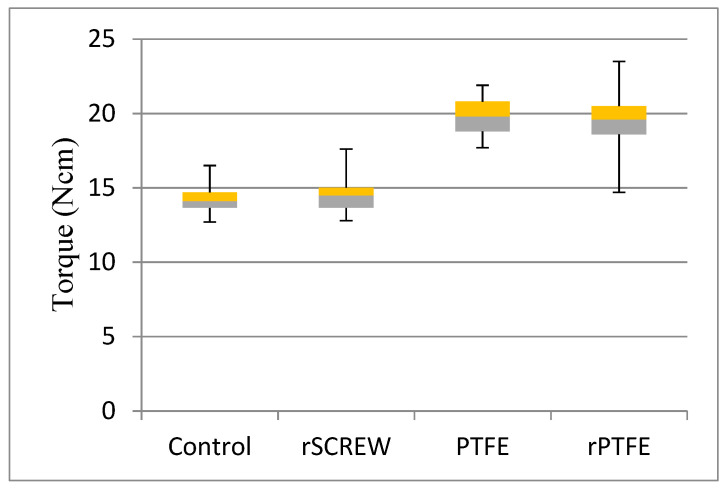
Boxplot of RTVs for control (*n* = 15) and experimental groups (*n* = 15).

No abutment mobility or screw fracture was perceptible to the touch or to the naked eye after the series of 300,000 cycles. The range of values obtained were higher in groups where PTFE tape was used (PTFE (17.7–21.9 Ncm) and rPTFE (14.6–23.5 Ncm) groups) than in the SCREW group (12.7–16.5 Ncm) and rSCREW group (12.8–17.6 Ncm). The mean RTVs were 14.46 ± 0.99 Ncm for the SCREW group (control), 14.42 ± 1.22 Ncm for the rSCREW group, 19.97 ± 1.16 Ncm for the PTFE group, and 19.13 ± 2.01 Ncm for the rPTFE group (Figure 6). 

The values found with the Tukey’s test at a significance level of *p* = 0.0001 confirmed the existence of significant differences between the RTVs for the control and rSCREW versus rPTFE and PTFE groups. No significant differences were found between the control group and rSCREW and rPTFE groups compared to the PTFE group (Table 1).

## 4. Discussion

The present study aimed to assess the efficacy of employing PTFE wrapping of the prosthetic screws of implant-supported single crowns to lower the risk of SL and to solve this complication when it appears. By accepting the assumption that the RTV is a measure of the remaining preload in the abutment screw [9,18,19,35], the increase of postload reverse torque of two experimental groups (PTFE and rPTFE) in the present study confirms the research hypothesis, since the existence of significant differences between the RTVs for the control and experimental groups were found.

SL has been reported as the most frequent mechanical complication of implant restorations [36]. The gravity of SL as a biomechanical problem is attested to by the number of studies assessing the factors that may affect its appearance. The methods used in those studies may condition the findings, however, hampering the establishment of general conclusions, such as the degree to which each factor determines the onset of loosening. SL may be caused by inadequate tightening torque, settling of implant components, inappropriate implant position, inadequate occlusal scheme or crown anatomy, poorly fitting frameworks, improper screw design/material, type of implant connection, type of abutment, and heavy occlusal forces [37]. To overcome screw loosening and joint instability, many technical solutions have been suggested—for example, type of implant connection, screw material/design, type of abutment, and number of load cycles.

This lack of methodological uniformity is visible, for instance, in the type of implant connection studied or how cyclic loads are applied. The magnitude, frequency, and number of loads reported in the literature also vary widely, with the number of cycles ranging from 16,667 to 5,000,000, the force from 20 to 420 N, and the frequency from 1 to 30 Hz [34,38,39,40]. The number of cycles (300,000) per sample applied in this study was used as representative of 1 year of mastication [35], whereas the reference for the force used was the range found in the literature for chewing force: 143–330 N [41].

The type of connection is indisputably one of the factors most frequently analyzed in previous studies [20,38]. Most concluded that the Morse taper retains preload more effectively than the external hex [35] and, to a lesser extent, even the internal hex connection [20] because it reduces micromovements, thus enhancing screw stability and stress dissipation [19]. Some authors’ recommendation to use internal hex and Morse taper connections for this reason, particularly in single-crown implants, is reasonable. That procedure would not eradicate the problem entirely, however. Moreover, one must take into account the amount of external hex implants already placed in patients’ mouths that are prone to such loosening. The present study consequently addressed the effect PTFE tape wrapping on external hexagon connection implants. The type of implant connection was intentionally chosen as the least favorable circumstance, as earlier studies found that type of connection to exhibit the highest rate of screw loosening [42].

Despite the many prosthetic-design-related measures for lowering the incidence of this complication, such as centering occlusal contact, narrowing the buccolingual width of the crown, or shortening the cantilever, screw loosening rates continue to be high, particularly in externally connected single-crown implants [19,43,44]. However, it is not always possible to achieve such ideal conditions in the oral environment. The efficacy of such measures would therefore appear to be limited.

Other recommendations have also been put forward to reduce SL by perfecting tightening techniques, such as screw readjustment 10 min after tightening to enhance screw stability [45,46], but the efficacy of this technique has not been proven in previous research. Some studies conclude that retightening loosened screws induces a progressive decline in the abutment–implant RTV with each new use [47], which they attribute to a deformation-mediated loss of friction between screw and implant. They consequently recommend using a new screw wherever definitive prostheses are involved. That finding is pertinent to the present study, where for the experimental group with used screws wrapped in PTFE, the RTV not only failed to decline but actually rose by 32% relative to the control.

One of the main causes of SL is the settling effect. It has been reported that 2–10% of the initial preload is lost without being subjected to any type of load as a result of the settling effect [48]. Preload depends of the following factors: (1) torque applied, which influences the screw head friction, the thread friction, and the elastic/plastic deformation of the screw; (2) screw head geometry, which influences the screw head friction; and (3) the screw and abutment material, which influences the level of grip. From a biomechanical point of view, the applied torque determines the preload, which in turn is responsible for the clamping forces; such forces arise from both attrition between opposing surfaces (screw and fixture) and elastic/plastic deformation occurring on the mating structures [49]. The application of cyclic loads was to simulate the dynamic conditions found in the oral cavity. Due to micromovement as a result of functional or parafunctional load [47], the use of PTFE tape might play a role in decreasing SL. The fact that the experimental group RTV showed better results than the control group could mean that the use of PTFE tape could reduce the loss of preload and maintain the initial force applied to the abutment screws. Further investigations are required to verify the effects of the technique planted in the present study.

Screw design unquestionably also has a heavy impact on SL [43]. The presence on the marketplace of screws especially designed to prevent loosening, such as those plated in gold, stands as proof of the importance attached to the problem. While some studies report higher reverse torque values [19,33] with gold-plated screws [43,50,51], no consensus has been reached in this regard [18,39]. The high cost of gold-plated screws is a significant drawback that both precludes their routine use and determines their relegation to specific clinical situations such as the loosening of a titanium screw. PTFE tape instead is an inexpensive and readily available material that would not be subject to that disadvantage.

In the literature review on the occasion of this study, only one paper was found that analyzed PTFE-plated screws [25]. Four different coated abutment screws groups were used (TiC, TiCN, Teflon, and Parylene) to clarify the influence of the coating material on abutment screw stability. The specimens were submitted to a series of three mechanical tests: (1) measuring the torque to unscrew a previously tight screw, (2) tightening and retightening six times the same screw, and (3) cyclic loading of the dental implant system. The study showed that RTVs were found not to rise with PTFE but actually to decline after the cyclic load measurement was performed. Nonetheless, the authors coated the screws with PTFE, an approach that differed from the sheathing procedure adopted here.

Further to the present findings, wrapping screws in PTFE would appear to be a convenient and viable solution for loosened screws, as it affords up to 32% higher reverse torque values than new screws, with the added advantage of its comparatively low cost. Further clinical studies are needed to determine whether this technique can effectively prevent and solve screw loosening. Furthermore, wrapping prosthetic screws with PTFE tape could be a new approach specially indicated to solve screw loosening, replacing gold as a plating material.

## 5. Conclusions

This in vitro study showed that wrapping screws in three windings of 100 µm polytetrafluorethylene tape raised the reverse torque value of loosened screws. Subject to the limitations of such research, this procedure may be deemed to effectively lower the likelihood of screw loosening in future implants and to be a viable solution when it appears in those now in place.

## Figures and Tables

**Table 1:** Mean RTV, standard deviation (SD), and statistical significance (*p*) between SCREW (control) and experimental groups.

Group	Specimen No.	Mean RTV (Ncm)	SD	*p*
SCREW (control)	15	14.46	±0.99	
rSCREW	15	14.42	±1.22	*p* = 0.98
PTFE	15	19.97	±1.17	*p* = 0.001
rPTFE	15	19.13	±2.01	*p* = 0.001

RTV: reverse torque value; SD: standard deviation.

## Data Availability

The data presented in this study are available on request from the corresponding author.

## References

[1] Dias E.C., Bisognin E.D., Harari N.D., Machado S.J., Da Silva C.P., Soares G.D., Vidigal G.M. (2012). Evaluation of implant-abutment microgap and bacterial leakage in five external-hex implant systems: An in vitro study. Int. J. Oral Maxillofac. Implants.

[2] Gracis S., Michalakis K., Vigolo P., Vult von Steyern P., Zwahlen M., Sailer I. (2012). Internal vs. external connections for abutments/reconstructions: A systematic review. Clin. Oral Implants Res..

[3] Pimentel A.C., Manzi M.R., Sartori S.G., Da Graça Naclerio-Homem M., Sendyk W.R. (2014). In vivo effectiveness of silicone gel sheets as barriers at the inner microgap between a prosthetic abutment and an external-hexagon implant platform. Int. J. Oral Maxillofac. Implants.

[4] Ramos M.B., Pegoraro L.F., Takamori E., Coelho P.G., Silva T.L., Bonfante E.A. (2014). Evaluation of UCLA implant-abutment sealing. Int. J. Oral Maxillofac. Implants.

[5] Koutouzis T., Neiva R., Nonhoff J., Lundgren T. (2013). Placement of implants with platform-switched Morse taper connections with the implant-abutment interface at different levels in relation to the alveolar crest: A short-term (1-year) randomized prospective controlled clinical trial. Int. J. Oral Maxillofac. Implants.

[6] Gaviria L., Salcido J.P., Guda T., Ong J.L. (2014). Current trends in dental implants. J. Korean Assoc. Oral Maxillofac. Surg..

[7] Kanneganti K., Vinnakota D., Pottem S., Pulagam M. (2018). Comparative effect of implant-abutment connections, abutment angulations, and screw lengths on preloaded abutment screw using three-dimensional finite element analysis: An in vitro study. J. Indian Prosthodont. Soc..

[8] Sailer I., Mühlemann S., Zwahlen M., Hämmerle C.H., Schneider D. (2012). Cemented and screw-retained implant reconstructions: A systematic review of the survival and complication rates. Clin. Oral Implants Res..

[9] Kim S.K., Koak J.Y., Heo S.J., Taylor T.D., Ryoo S., Lee S.Y. (2012). Screw loosening with interchangeable abutments in internally connected implants after cyclic loading. Int. J. Oral Maxillofac. Implants.

[10] Jemt T., Laney W.R., Harris D., Henry P.J., Krogh P.H., Polizzi G., Zarb G.A., Herrmann I. (1991). Osseointegrated implants for single tooth replacement: A 1-year report from a multicenter prospective study. Int. J. Oral Maxillofac. Implants.

[11] Naert I., Quirynen M., Van Steenberghe D., Darius P. (1992). A study of 589 consecutive implants supporting complete fixed prostheses. Part II: Prosthetic aspects. J. Prosth. Dent..

[12] Lekholm U., Gunne J., Henry P., Higuchi K., Linden U., Bergstrom C. (1999). Survival of the Branemark implant in partially edentulous jaws: A 10-year prospective multicenter study. Int J. Oral Maxillofac. Implants.

[13] Lindhe J., Meyle J. (2008). Peri-implant diseases: Consensus report of the sixth European workshop on periodontology. J. Clin. Periodontol..

[14] Canallatos J.E., Hobbs G.R., Bryington M.S., Dye B.D. (2020). The effect of implant prosthesis complications on patient satisfaction. J. Prosthet. Dent..

[15] Bickford J.H. (1995). An Introduction to the Design and Behavior of Bolted Joints.

[16] Do Nascimento C., Miani P.K., Pedrazzi V., Gonçalves R.B., Ribeiro R.F., Faria A.C. (2012). Leakage of saliva through the implant‑abutment interface: In vitro evaluation of three different implant connections under unloaded and loaded conditions. Int. J. Oral Maxillofac. Implants.

[17] Panza L., Boscatto N., Cury A.A. (2010). Evaluation of pre‑tightening in abutments and prosthetic screws on different implant connections. Rev. Odontol. Cienc..

[18] Tsuge T., Hagiwara Y. (2009). Influence of lateral‑oblique cyclic loading on abutment screw loosening of internal and external hexagon implants. Dent. Mater. J..

[19] Ha C.Y., Lim Y.J., Kim M.J., Choi J.H. (2011). The influence of abutment angulation on screw loosening of implants in the anterior maxilla. Int. J. Oral Maxillofac. Implants.

[20] Feitosa P.C., De Lima A.P., Silva-Concílio L.R., Brandt W.C., Neves A.C. (2013). Stability of external and internal implant connections after a fatigue test. Eur. J. Dent..

[21] Martin W.C., Woody R.D., Miller B.H., Miller A.W. (2001). Implant abutment screw rotations and preloads for four different screw materials and surfaces. J. Prosthet. Dent..

[22] Shenava A. (2013). Failure mode of implant abutment connections: An overview. J. Dent. Med. Sci.

[23] Kano S.C., Binon P.P., Curtis D.A. (2007). A classification system to measure the implant-abutment microgap. Int. J. Oral Maxillofac. Implants.

[24] Covani U., Ricci M., Tonelli P., Barone A. (2013). An evaluation of new designs in implant-abutment connections: A finite element method assessment. Implant Dent..

[25] Elias C.N., Figueira D.C., Rios P.R. (2006). Influence of the coating material on the loosing of dental implant abutment screw joints. Mater. Sci. Eng..

[26] Theoharidou A., Petridis H.P., Tzannas K., Garefis P. (2008). Abutment screw loosening in single-implant restorations: A systematic review. Int. J. Oral Maxillofac. Implants.

[27] Pardal-Peláez B., Montero J. (2017). Preload loss of abutment screws after dynamic fatigue in single implant-supported restorations. A systematic review. J. Clin. Exp. Dent..

[28] Hess T.A. (2014). A technique to eliminate subgingival cement adhesion to implant abutments by using polytetrafluoroethylene tape. J. Prosthet. Dent..

[29] Moráguez O., Belser U. (2010). The use of polytetrafluoroethylene tape for the management of screw access channels in implant-supported prostheses. J. Prosthet. Dent..

[30] Cavalcanti A.G., Fonseca F.T., Zago C.D., Brito R., Franca F.M. (2016). Efficacy of gutta-percha and polytetrafluoroethylene tape to microbiologically seal the screw access channel of different prosthetic implant abutments. Clin. Implant Dent. Relat. Res..

[31] Bulaqi H.A., Barzegar A., Paknejad M., Safari H. (2019). Assessment of preload, remaining torque, and removal torque in abutment screws under different frictional conditions: A finite element analysis. J. Prosthet. Dent..

[32] Burguete R.L., Johns R.B., King T., Patterson E.A. (1994). Tightening characteristics for screwed joints in osseointegrated dental implants. J. Prosthet. Dent..

[33] Stüker R.A., Teixeira E.R., Beck J.C., Costa N.P. (2008). Preload and torque removal evaluation of three different abutment screws for single standing implant restorations. J. Appl. Oral Sci..

[34] Park J.K., Choi J.U., Jeon Y.C., Choi K.S., Jeong C.M. (2010). Effects of abutment screw coating on implant preload: Preload on screw coating and connection types. J. Prosthodont..

[35] Khraisat A., Abu-Hammad O., Dar-Odeh N., Al-Kayed A.M. (2004). Abutment screw loosening and bending resistance of external hexagon implant system after lateral cyclic loading. Clin. Implant Dent. Relat. Res..

[36] Goodacre C.J., Bernal G., Rungcharassaeng K., Kan J.Y. (2003). Clinical complications with implants and implant prostheses. J. Prosthet. Dent..

[37] Schwarz M.S. (2000). Mechanical complications of dental implants. Clin. Oral Implants Res..

[38] Siadat H., Pirmoazen S., Beyabanaki E., Alikhasi M. (2015). Does abutment collar length affect abutment screw loosening after cyclic loading. J. Oral Implantol..

[39] Yao K.T., Kao H.C., Cheng C.K., Fang H.W., Yip S.W., Hsu M.L. (2012). The effect of clockwise and counterclockwise twisting moments on abutment screw loosening. Clin. Oral Implants Res..

[40] Lee C.K., Karl M., Kelly J.R. (2009). Evaluation of test protocol variables for dental implant fatigue research. Dent. Mater..

[41] Mericske-Stern R., Zarb G.A. (1996). In vivo measurements of some functional aspects with mandibular fixed prostheses supported by implants. Clin. Oral Implants Res..

[42] Jorge J., Barao V., Delben J., Assuncao W. (2013). The role of implant/abutment system on torque maintenance of retention screws and vertical misfit of implant-supported crowns before and after mechanical cycling. Int. J. Oral Maxillofac. Implants.

[43] Piermatti J., Yousef H., Luke A., Mahevich R., Weiner S. (2006). An in vitro analysis of implant screw torque loss with external hex and internal connection implant systems. Implant Dent..

[44] Coppedê A.R., Faria A.C., De Mattos Mda G., Rodrigues R.C., Shibli J.A., Faria Ribeiro R. (2013). Mechanical comparison of experimental conical-head abutment screws with conventional flat-head abutment screws for external-hex and internal Tri-channel implant connections: An In Vitro evaluation of loosening torque. Int. J. Oral Maxillofac. Implants.

[45] Shin H.M., Huh J.B., Yun M.J., Jeon Y.C., Chang B.M., Jeong C.M. (2014). Influence of the implant-abutment connection design and diameter on the screw joint stability. J. Adv. Prosthodont..

[46] Siamos G., Winkler S., Boberick K.G. (2002). The relationship between implant preload and screw loosening on implant-supported prostheses. J. Oral Implantol..

[47] Weiss E., Kozak D., Gross M. (2000). Effect of repeated closures on opening torque values in seven abutment-implant systems. J. Prosthet. Dent..

[48] Bakaeen L.G., Winkler S., Neff P.A. (2001). The effect of implant diameter, restoration design, and occlusal table variations on screw loosening of posterior single-tooth implant restorations. J. Oral Implantol..

[49] McGlumphy E.A., Mendel D.A., Holloway J.A. (1998). Implant screw mechanics. Dent. Clin. N. Am..

[50] Zipprich H., Weigl P., Ratka C., Lange B., Lauer H.C. (2018). The micromechanical behavior of implant-abutment connections under a dynamic load protocol. Clin. Implant Dent. Relat. Res..

[51] Lang L.A., Kang B., Wang R., Lang B. (2003). Finite element analysis to determine implant preload. J. Prosthet. Dent..

